# New Doc on the Block: Scoping Review of AI Systems Delivering Motivational Interviewing for Health Behavior Change

**DOI:** 10.2196/78417

**Published:** 2025-09-16

**Authors:** Zev Karve, Jacob Calpey, Christopher Machado, Michelle Knecht, Maria Carmenza Mejia

**Affiliations:** 1 Schmidt College of Medicine Florida Atlantic University Boca Raton, FL United States

**Keywords:** artificial intelligence, AI, motivational interviewing, large language models, behavior change, digital health

## Abstract

**Background:**

Artificial intelligence (AI) is increasingly used in digital health, particularly through large language models (LLMs), to support patient engagement and behavior change. One novel application is the delivery of motivational interviewing (MI), an evidence-based, patient-centered counseling technique designed to enhance motivation and resolve ambivalence around health behaviors. AI tools, including chatbots, mobile apps, and web-based agents, are being developed to simulate MI techniques at scale. While these innovations are promising, important questions remain about how faithfully AI systems can replicate MI principles or achieve meaningful behavioral impact.

**Objective:**

This scoping review aimed to summarize existing empirical studies evaluating AI-driven systems that apply MI techniques to support health behavior change. Specifically, we examined the feasibility of these systems; their fidelity to MI principles; and their reported behavioral, psychological, or engagement outcomes.

**Methods:**

We systematically searched PubMed, Embase, Scopus, Web of Science, and Cochrane Library for empirical studies published between January 1, 2018, and February 25, 2025. Eligible studies involved AI-driven systems using natural language generation, understanding, or computational logic to deliver MI techniques to users targeting a specific health behavior. We excluded studies using AI solely for training clinicians in MI. Three independent reviewers screened and extracted data on study design, AI modality and type, MI components, health behavior focus, MI fidelity assessment, and outcome domains.

**Results:**

Of the 1001 records identified, 15 (1.5%) met the inclusion criteria. Of these 15 studies, 6 (40%) were exploratory feasibility or pilot studies, and 3 (20%) were randomized controlled trials. AI modalities included rule-based chatbots (9/15, 60%), LLM-based systems (4/15, 27%), and virtual or mobile agents (2/15, 13%). Targeted behaviors included smoking cessation (6/15, 40%), substance use (3/15, 20%), COVID-19 vaccine hesitancy, type 2 diabetes self-management, stress, mental health service use, and opioid use during pregnancy. Of the 15 studies, 13 (87%) reported positive findings on feasibility or user acceptability, while 6 (40%) assessed MI fidelity using expert review or structured coding, with moderate to high alignment reported. Several studies found that users perceived the AI systems as judgment free, supportive, and easier to engage with than human counselors, particularly in stigmatized contexts. However, limitations in empathy, safety transparency, and emotional nuance were commonly noted. Only 3 (20%) of the 15 studies reported substantially significant behavioral changes.

**Conclusions:**

AI systems delivering MI show promise for enhancing patient engagement and scaling behavior change interventions. Early evidence supports their usability and partial fidelity to MI principles, especially in sensitive domains. However, most systems remain in early development, and few have been rigorously tested. Future research should prioritize randomized evaluations; standardized fidelity measures; and safeguards for LLM safety, empathy, and accuracy in health-related dialogue.

**Trial Registration:**

OSF Registries 10.17605/OSF.IO/G9N7E; https://osf.io/g9n7e

## Introduction

### Background

Health behavior change, such as quitting smoking, increasing physical activity, or adhering to prescribed treatments, is among the most impactful yet persistently difficult challenges in preventive and chronic disease care. Motivational interviewing (MI), a structured, patient-centered counseling approach that enhances intrinsic motivation by resolving ambivalence, has been shown to be effective across a wide range of settings, including primary care, mental health, addiction treatment, and chronic disease management [1,2].

Despite MI’s strong evidence base, its widespread implementation in clinical settings remains limited. Barriers include time constraints, inadequate clinician training, and limited reimbursement mechanisms [3]. As a result, there is a growing interest in leveraging digital tools to scale the delivery of MI. Artificial intelligence (AI) offers novel opportunities to deliver MI techniques through automated, scalable technologies, including rule-based agents, natural language processing (NLP) systems, and large language models (LLMs).

AI systems designed for MI delivery may take the form of chatbots, mobile apps, or embodied virtual agents that simulate empathic, supportive dialogue. Some systems use scripted logic to guide conversations, while others rely on advanced generative models such as GPT-4 to dynamically respond to user input. Recent innovations suggest that such tools can simulate core MI components, including open-ended questions, reflective listening, affirmations, and elicitation of change talk [4-6]. These features may enhance user engagement by providing consistent, nonjudgmental, and round-the-clock interactions, particularly in stigmatized behavioral health contexts [7-9].

However, while studies increasingly report high feasibility and user acceptability of AI-driven MI tools, few have rigorously evaluated their fidelity to MI principles or their impact on behavioral outcomes [10-12]. MI fidelity—the extent to which systems demonstrate MI-consistent behaviors such as empathy, autonomy support, and evocation—remains underexplored. Assessing fidelity is especially challenging in the context of AI, where interactions may lack relational nuance or contextual sensitivity. Historically, fidelity assessment has relied on structured human-coded frameworks such as the Motivational Interviewing Treatment Integrity (MITI) scale or the Motivational Interviewing Skills Code, both of which are resource intensive and difficult to implement at scale [13,14].

This review also responds to recent calls for greater clarity about how MI techniques are being operationalized in digital systems [5,11]. As prior reviews have noted, many interventions include motivational “elements” without specifying whether and how they align with formal MI practices [7,15]. In response, we mapped the specific MI techniques (eg, open-ended questions, complex reflections, affirmations, ruler talk, and change talk elicitation) used in each study. We also examined whether fidelity was assessed using formal methods and expert review or inferred through study design.

### Objectives

To address these knowledge gaps, we conducted a scoping of empirical studies evaluating AI-driven systems designed to deliver MI to patients. We aimed to characterize how these systems apply MI principles and evaluate their feasibility, fidelity, and behavioral impact. Our research questions were as follows: (1) How are AI-driven systems, including chatbots and LLMs, being used to deliver MI? (2) What is known about their feasibility and usability? (3) To what extent do they demonstrate fidelity to MI principles? (4) What behavioral or psychological outcomes have been reported?

## Methods

This scoping review was conducted in accordance with the JBI methodological framework for scoping reviews and reported in line with the PRISMA-ScR (Preferred Reporting Items for Systematic Reviews and Meta-Analyses extension for Scoping Reviews) checklist [16,17]. The review protocol was registered on OSF on May 30, 2025, and is publicly available [18].

### Search Strategy

An initial exploratory search of MEDLINE (PubMed) was conducted to refine terminology and develop an appropriate strategy. A medical librarian (MK) with expertise in scoping review methodology developed the final search strategy using a combination of free-text terms and controlled vocabulary (eg, Medical Subject Headings [MeSH]) covering “artificial intelligence,” “large language models,” “natural language processing,” “chatbots,” “virtual agents,” “motivational interviewing,” “behavior change,” and “patient counseling.” The full search strategy is presented in Multimedia Appendix 1. A comprehensive search was then conducted across 5 databases: PubMed, Embase, Cochrane Library, Scopus, and Web of Science. Searches were limited to studies published between January 1, 2018, and February 25, 2025.

The 2018 start date was chosen to align with the emergence of scalable AI systems capable of generating language through deep learning and neural models, including early LLMs and transformer-based architectures. While foundational research in MI-related chatbots predates this period [19,20], the post-2018 literature reflects a technological turning point in conversational AI, with increased relevance to modern health interventions.

### Inclusion and Exclusion Criteria

We included empirical studies of any design that evaluated an AI system delivering MI directly to individuals, targeting specific health behavior (eg, smoking cessation, substance use, chronic disease self-care, or stress management). To capture the evolving nature of these technologies, we included both complete MI interventions and systems designed to generate or evaluate discrete MI components, such as open-ended questions, reflections, or change talk statements. This review includes both full-scale, AI-delivered MI interventions and systems developed to produce only discrete MI components, such as reflective utterances or open-ended questions. These limited-functionality systems were included to explore how MI techniques are being tested or approximated in emerging AI tools. This approach aligns with our aim to understand how MI is being operationalized across a spectrum of AI sophistication, from fully web-based conversational agents to single-turn utterance generators.

The inclusion and exclusion criteria are presented in Textbox 1.

**Inclusion and exclusion criteria.**

**Inclusion criteria**

- Studies that used artificial intelligence (AI) systems incorporating natural language generation, understanding, or computational logic to simulate motivational interviewing (MI) techniques
- Studies that delivered the intervention directly to end users (eg, patients, college students, or community members)
- Studies that evaluated system functionality, MI fidelity, or behavioral and psychological outcomes

**Exclusion criteria**

- Studies that focused solely on using AI for MI training or clinician education
- Studies that did not involve direct AI-user interaction
- Studies that described conceptual frameworks or prototypes without any empirical testing

### Study Selection

After deduplication, all titles and abstracts were screened independently by 3 reviewers (ZK, CM, and JC) using Covidence (Veritas Health Innovation), a web-based software platform designed to support systematic and scoping review workflows. Covidence facilitates blinded screening, conflict resolution, and full-text review. Full texts of potentially relevant articles were assessed independently by at least 2 reviewers, with discrepancies resolved through consensus or adjudication by a senior reviewer (MCM).

### Data Extraction and Synthesis

A customized data extraction tool (Multimedia Appendix 2) was developed to capture relevant study characteristics, including publication year, study design, sample size, targeted health behavior, user population, AI modality (eg, chatbot, mobile app, or virtual agent), AI type (eg, rule-based or LLM-based), MI techniques used, outcome domains, key findings, and author-reported limitations. We also documented whether studies evaluated MI fidelity and, if so, how it was assessed, such as through expert raters, structured coding frameworks (eg, the MITI scale), or automated tools.

We defined MI fidelity as the extent to which an AI system demonstrates alignment with core MI principles, including empathy, reflective listening, collaboration, evocation, and autonomy support. Fidelity was considered “assessed” only when a structured approach, such as expert evaluation, predefined rating criteria, or a validated coding scheme, was explicitly used to evaluate AI outputs.

We also mapped the specific MI techniques incorporated into each system (a brief glossary is included in Multimedia Appendix 2). These techniques included open-ended questions, affirmations, reflections, elicitation of change talk, and ruler talk. The presence of these techniques was based on author descriptions, illustrative examples, or discernible features in the conversational design. This mapping allowed us to compare how different AI systems operationalized MI—whether through static scripts or generative AI—and identify gaps in technique application and depth.

Given the increasing use of generative AI models in health communication, we included a dedicated extraction item to assess whether each study addressed safety considerations, such as the risk of misinformation, inappropriate content generation, or ethical use. We noted whether authors described guardrails, expert oversight, or content validation mechanisms to mitigate known risks associated with LLMs. This reflects recent recommendations for responsible AI deployment in behavioral health and clinical settings.

Outcome domains were categorized into three primary groups to facilitate synthesis: (1) behavioral outcomes (eg, smoking cessation and reduced substance use), (2) psychological outcomes (eg, confidence, readiness to change, and perceived empathy), and (3) engagement outcomes (eg, usability, user satisfaction, and perceived alliance).

Due to the heterogeneity in AI architecture, study design, evaluation methods, and outcome measures, the results were synthesized narratively. Descriptive statistics were used where appropriate. The final synthesis was structured around the following three dimensions: (1) feasibility and usability of AI-driven MI systems; (2) fidelity to MI principles; and (3) reported behavioral, psychological, or engagement outcomes.

## Results

### Study Characteristics

**Figure 1:**
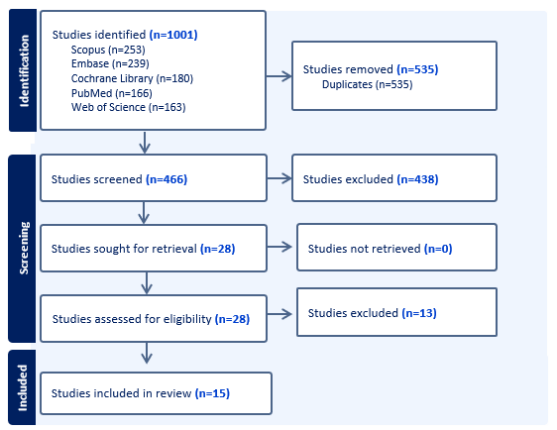
PRISMA (Preferred Reporting Items for Systematic Reviews and Meta-Analyses) flowchart illustrating the identification, screening, eligibility assessment, and inclusion of studies in the scoping review on AI-delivered motivational interviewing.

Our search identified 1001 articles, of which 535 (53.45%) were duplicates, while 438 (43.76%) were excluded after title and abstract screening (Figure 1). A full-text review of the remaining 28 studies yielded 15 (54%) eligible studies [4-11,21-27]. Table 1 presents a summary of the included studies, highlighting their design, populations, study purpose, key outcomes, and safety considerations. The complete dataset with additional details is provided in Multimedia Appendix 3 [4-11,21-27].

Most of the studies (14/15, 93%) were published between 2020 and 2025 and represented early-phase evaluations, such as iterative development, pilot testing, or prototype assessments. Of the 15 studies, 3 (20%) used randomized controlled trial (RCT) designs: He et al [22] conducted a between-participants RCT comparing an MI-style chatbot to a neutral control for smoking cessation, He et al [23] used a mixed methods RCT to assess an MI chatbot versus a confrontational counseling chatbot, and Leeuwis and He [10] performed a between-participants RCT evaluating MI versus confrontational counseling chatbot interactions with attention to psychological moderators [10]. The remaining studies (12/15, 80%) used single-arm trials, mixed methods, qualitative approaches, or small-scale pilots.

Study populations ranged from general adult users to patients with specific conditions. A few of the studies (4/15, 27%) recruited college students or individuals undergoing health screening. Sample sizes varied widely, from small usability cohorts (eg, n=10) to larger RCTs with more than 200 participants. The most common targeted behavior was smoking cessation (6/15, 40%), followed by other substance use reduction (eg, opioid use during pregnancy and alcohol; 3/15, 20%), COVID-19 vaccine hesitancy (1/15, 7%), stress management (1/15, 7%), diabetes self-management (1/15, 7%), treatment seeking for eating disorders (1/15, 7%), physical activity and nutrition (1/15, 7%), and alcohol-associated cirrhosis (1/15, 7%). Participant populations varied across studies and included general adults, individuals with chronic diseases, and college students.

AI modalities included chatbots (12/15, 80%), virtual agents (2/15, 13%), and mobile apps (1/15, 7%). AI types ranged from rule-based systems to LLMs such as GPT-3.5 and GPT-4. All studies used AI to simulate MI techniques to some degree, although their fidelity and evaluation rigor varied. Only a minority of the included studies (2/15, 13%) explicitly discussed safety measures related to LLM-generated content. Herbert et al [6] and Kumar et al [5] noted expert review processes to ensure safety and alignment with clinical guidance. However, most of the studies (13/15, 87%) did not describe specific strategies to prevent hallucinations, misinformation, or inappropriate dialogue generation.

**Table 1:** Characteristics of included studies evaluating artificial intelligence (AI)–delivered motivational interviewing (MI) across health domains.

Study (year)	Study design	Sample size, n	Study population	Study purpose	Outcomes	Addressed safety concerns
Almusharraf et al [21] (2020)	Iterative development	121	Adult daily smokers (age: 16-60 y) from the United Kingdom, the United States, and Canada; recruited via Prolific; no comparison group	To design and iteratively train a chatbot using MI techniques to engage ambivalent smokers	Enjoyment, engagement, and readiness to quit	Not explicitly addressed
Brown et al [4] (2023)	Iterative development	349	Adult daily smokers (age: ≥18 y) recruited via Prolific	To develop a chatbot using GPT-2-XL to deliver MI-style generative reflections	Readiness, confidence, and user experience	Not explicitly addressed
He et al [22] (2022)	RCT^a^ (comparative)	172	Daily smokers (age: ≥18 y); MI vs neutral chatbot	To evaluate whether an MI-style chatbot could improve user engagement and therapeutic alliance compared to a neutral chatbot	Engagement, therapeutic alliance, intention to quit, and perceived empathy	Not explicitly addressed
He et al [23] (2024)	Mixed methods RCT	287	Adult daily smokers (age: ≥18 y) recruited via Prolific; MI chatbot vs confrontational counseling chatbot	Compare MI chatbot vs confrontational counseling chatbot on smoking cessation motivation and alliance	Motivation to quit, perceived empathy, chatbot alliance, and emotional responses	Not explicitly addressed
Herbert et al [6] (2025)	Exploratory evaluation	N/A^b^ (expert ratings)	Expert reviewers assessing AI responses related to OUD^c^ during pregnancy	Evaluate safety and MI alignment of GPT-4 chatbot content	Safety and MI alignment (expert rated)	Expert validation for safety and MI consistency
Kumar et al [5] (2024)	Algorithm development and validation	50 transcripts (150 responses)	Expert reviewers evaluated AI outputs	To generate and validate backward-looking complex reflections using GPT-4 for MI chatbot	Acceptability of backward-looking complex reflections generated; interrater agreement	Expert validation for reflection quality
Leeuwis and He [10] (2023)	RCT (comparative)	233	Smokers (age: ≥18 y) recruited online; MI chatbot vs confrontational counseling chatbot	To compare effects of an MI chatbot vs a confrontational counseling chatbot on smoking cessation intent, moderated by self-efficacy and autonomy	Quit intention, user satisfaction, and therapeutic alliance	Not explicitly addressed
Li et al [11] (2024)	Pilot feasibility	32	Adults in Hong Kong who were vaccine hesitant (mostly aged 18-29 y, predominantly female)	To develop and pilot a theory-based AI chatbot (“Auricle”) integrating MI to reduce COVID-19 vaccine hesitancy	Vaccine literacy, confidence, readiness, and intention	Not explicitly addressed
Meywirth et al [24] (2024)	Prototype (design science)	11	Adults in Germany interested in improving fitness and nutrition; varying LLM^d^ familiarity	To design and evaluate an LLM-based MI intervention for lifestyle behavior change	Engagement, goal setting, and perceived support	Expert review for MI alignment and safety
Park et al [25] (2019)	Qualitative	30 (10 interviewed)	College students in South Korea	To design and test a chatbot delivering a brief MI-based stress management conversation	User feedback and design insights	Safety concern acknowledged; no formal evaluation
Prochaska et al [7] (2021)	Single-arm pretest-posttest pilot	101	Adults (age: 18-65 y) with problematic substance use, recruited online	To evaluate feasibility, usability, and preliminary effects of an MI-informed digital agent (Woebot for the treatment of substance use disorders)	Usability, craving reduction, engagement, and confidence	Not addressed
Shah et al [26] (2022)	Iterative usability	21	Individuals who screened positive for eating disorders	To develop and refine a chatbot (“Alex”) pairing with eating disorder screening to encourage treatment engagement	SUS^e^; the Usefulness, Satisfaction, and Ease of Use questionnaire; and qualitative feedback	Not addressed
Steenstra et al [27] (2024)	3-part evaluation	40 (study 1), 12 (study 2), and 8 (study 3)	Lay participants and MI experts role-playing as clients	Evaluate whether a GPT-4–powered virtual agent can replicate MI for alcohol use counseling	MI competency, safety, usability, and therapeutic alliance	Not addressed
Yeo et al [9] (2024)	Pilot feasibility	24	Patients with alcohol-associated cirrhosis	Evaluate feasibility and usability of an AI conversational agent in VR^f^ setting	Feasibility and usability	Safety risks acknowledged (LLM-generated content)
Yoon et al [8] (2024)	Qualitative	33	Patients in Singapore with type 2 diabetes mellitus, aged ≥40 y	Explore acceptability and preferences for app-based MI to support diabetes self-management	Acceptability and user feedback on MI features	Not addressed

^a^RCT: randomized controlled trial.

^b^N/A: not applicable.

^c^OUD: opioid use disorder.

^d^LLM: large language model.

^e^SUS: System Usability Scale.

^f^VR: virtual reality.

### Feasibility and Usability

All 15 studies reported that AI systems simulating MI were feasible to implement and generally acceptable to users. Interventions were often described as technically stable, user-friendly, and consistently structured, with several highlighting the “judgment-free” tone, on-demand availability, and capacity for personalization as key facilitators of engagement; for example, in the studies by Brown et al [4] and Almusharraf et al [21], chatbots delivering MI content were associated with increased user readiness and confidence to quit smoking. Yeo et al [9] reported that 85% of participants with alcohol-associated cirrhosis rated their experience with a virtual reality–based MI agent as beneficial. In the study by Yoon et al [8], participants noted that the MI app facilitated self-reflection and behavior change, although some expressed a preference for human interaction.

Even systems in early development stages [11,24,25] were described positively for tone and ease of use, although participants frequently suggested the need for greater emotional nuance, goal tracking, or tailored resources to sustain motivation over time. Across studies, intuitive design and empathic conversation were seen as essential to system usability and acceptance.

### Fidelity to MI Principles

Of the 15 included studies, 6 (40%) formally assessed fidelity to MI principles through either structured coding frameworks or expert review of AI-generated content. These studies evaluated the degree to which systems demonstrated core MI elements such as empathy, reflective listening, evocation, and collaboration.

Brown et al [4] and Kumar et al [5] used expert raters to evaluate the quality of AI-generated reflections. Both reported that a high proportion of responses met MI-consistent standards. Notably, Kumar et al [5] found that 88% of AI-generated reflections met predefined MI criteria, demonstrating the potential of LLMs such as GPT-4 to generate responses that align with MI principles. The system applied by Brown et al [4] used dynamic conversational scaffolding, in which the AI builds on prior user responses to progressively guide the conversation. This was supported by context-specific generative reflections and pacing adjustments in subsequent work, Kumar et al [5] extended this approach to incorporate backward-looking reflections, where the chatbot synthesized earlier user input to guide dialogue based on prior responses

Herbert et al [6] similarly used expert review to evaluate outputs from a GPT-4–based chatbot providing opioid-related guidance and found favorable ratings for MI alignment and safety. In the study by Leeuwis and He [10], an experimental design compared MI and confrontational counseling chatbot versions. Although the study did not use formal coding tools such as the MITI scale, the comparison of alliance and autonomy outcomes between the arms provided indirect evidence of MI fidelity, particularly in how the MI condition influenced perceived empathy. He et al [23] conducted a mixed methods evaluation of a smoking cessation chatbot and included user-reported outcomes related to empathy and alliance. While fidelity was not assessed with a coding framework, structured comparisons between the MI and control arms offered insight into the chatbot’s ability to reflect MI principles. Finally, Steenstra et al [27] reported that the use of LLMs within virtual agents led to more MI-consistent dialogue, as confirmed by analysis of transcript excerpts and alignment with theoretical MI stages. However, this was not evaluated with a formal fidelity coding instrument.

By contrast, the remaining studies (9/15, 60) either referenced MI in theoretical terms or described the use of MI-consistent dialogue strategies (eg, open-ended questions and affirmations) but did not include formal fidelity assessment. This gap highlights the need for more rigorous evaluation of how well AI systems adhere to MI standards, particularly as these tools scale for clinical use.

Kumar et al [5] reported that 88% of AI-generated reflections met MI criteria, while Herbert et al [6] found that GPT-4, when tuned for MI, achieved high ratings for both safety and MI alignment from expert reviewers.

### Behavioral, Psychological, and Engagement Outcomes

Although all included studies aimed to promote health behavior change, only a few (5/15, 33%) reported direct behavioral outcomes. Prochaska et al [7] found statistically significant reductions in cravings and substance use after chatbot interaction. Meywirth et al [24] reported an increase in smoking cessation resource requests among chatbot users. He et al [22] observed stronger quitting intentions and greater engagement in the MI condition than in a neutral chatbot control.

Other studies evaluated psychological or motivational outcomes such as readiness to change, perceived empathy, self-efficacy, and engagement. Brown et al [4], Yoon et al [8], and Li et al [11] found improvements in user confidence, motivation, or openness to reflection. Several of the studies (4/15, 27%), including those by Shah et al [26] and Park et al [25], noted user appreciation for judgment-free communication, even in the absence of measurable behavioral change.

Engagement outcomes, including usability, acceptability, and interaction quality, were also commonly evaluated. Yeo et al [9] found that 85% of participants rated the virtual reality–based agent positively. Shah et al [26] and Park et al [25] highlighted user appreciation for the judgment-free tone of chatbot conversations. Features such as intuitive design, personalized prompts, and empathic dialogue contributed to stronger user engagement across multiple studies.

Importantly, no study measured long-term behavioral outcomes, and follow-up periods were often short or absent. Thus, while these studies support that AI systems can deliver motivational content and influence short-term cognitive and emotional precursors to change, the ability of these systems to drive sustained behavior change remains an open question.

### Summary in Relation to the Research Questions

This review found that AI systems, primarily chatbots, apps, and virtual agents, are increasingly designed to simulate MI, especially for smoking cessation, stress management, and chronic disease support. These systems are generally feasible, usable, and positively received. Of the 15 included studies, 6 (40%) assessed MI fidelity, using varied tools, and found moderate to strong alignment with MI principles. While a small number of studies (5/15, 33%) reported behavioral outcomes, most (10/15, 67%) focused on proximal psychological constructs, such as readiness or perceived empathy. Collectively, the evidence suggests that AI-based MI systems are promising but require further evaluation of long-term outcomes and more consistent fidelity assessment to fully understand their clinical utility. Table 2 maps each study according to its AI modality and type, MI techniques applied, fidelity assessment, and outcome domains, allowing for cross-study comparison aligned with our research questions.

**Table 2:** Summary of motivational interviewing (MI) techniques, fidelity assessment, and outcome domains across the included studies.

Study (year)	Health behavior	AI^a^ modality	AI type	MI techniques used	MI fidelity assessed^b^	Outcome domain^c^
Almusharraf et al [21] (2020)	Smoking cessation	Chatbot (text)	Rule-based+NLP^d^	Open-ended questions, reflections, and summaries	No (MI guided design only)	Psychological and engagement
Brown et al [4] (2023)	Smoking cessation	Chatbot (text)	LLM^e^ based (GPT-2 XL-3.5)	Open-ended questions, reflections, and affirmations	Yes (expert review of generative MI reflections)	Psychological and engagement
He et al [22] (2022)	Smoking cessation	Chatbot (text)	Rule-based	Reflections, open questions, change talk, and affirmations	No (MI vs control design; no formal coding)	Behavioral, psychological, and engagement
He et al [23] (2024)	Smoking cessation	Chatbot (text)	Rule-based	Reflections, open questions, and change talk	Yes (comparison with control+user feedback)	Psychological
Herbert et al [6] (2025)	Opioid use during pregnancy	Chatbot (text)	LLM-based (GPT-4)	Reflections, empathy, and autonomy support	Yes (expert rating of MI alignment)	Not specified
Kumar et al [5] (2024)	Smoking cessation	Chatbot (text)	LLM-based (GPT-4)	Reflections (backward-looking, complex)	Yes (structured checklist against MI elements)	Not specified
Leeuwis and He [10] (2023)	Smoking cessation	Chatbot (text)	Rule-based	Reflections, open questions, and affirmations	Yes (adapted CEMI^f^ fidelity scale)	Psychological
Li et al [11] (2024)	COVID-19 vaccine hesitancy	Chatbot (web based)	Rule-based+NLP	Open-ended questions, affirmations, reflections, and evocation	No (user acceptance only)	Psychological
Meywirth et al [24] (2024)	Physical activity and nutrition	Chatbot (text)	LLM based (ChatGPT with GPT-3.5)	Open-ended questions, affirmations, and reflections	No (guided by theory only)	Behavioral, engagement, and psychological
Park et al [25] (2019)	Stress management	Chatbot (text)	Rule-based	Open-ended questions and reflections	No (descriptive only)	Engagement (usability)
Prochaska et al [7] (2021)	Substance use reduction	Conversational agent	Rule-based	Reflections and change talk	No (fidelity referenced, not formally coded)	Behavioral, psychological, and engagement
Shah et al [26] (2022)	Treatment seeking for eating disorders	Chatbot (text)	Rule-based+NLP	Open-ended prompts, affirmations, and evocation	No (MI referenced; not assessed)	Engagement
Steenstra et al [27] (2024)	Alcohol misuse	Virtual agent	GPT-4	Open-ended questions, reflections, affirmations, and autonomy support	Yes (expert ratings using MI constructs; MITI-informed global items)	Psychological and engagement
Yeo et al [9] (2024)	Alcohol-associated cirrhosis	VR^g^+GPT-4 agent	Rule-based	Open-ended questions, affirmations, and reflections	No (user feedback only)	Engagement and psychological
Yoon et al [8] (2024)	Diabetes self-management	Mobile app	Rule-based+ML^h^	Open-ended questions, goal setting, and confidence rulers	No (perceptions reported; not formally rated)	Engagement and psychological

^a^AI: artificial intelligence.

^b^“Yes” indicates that fidelity to MI principles was evaluated using structured coding frameworks (eg, Motivational Interviewing Treatment Integrity [MITI]) or expert review of system outputs; “no” denotes that fidelity was not formally assessed, even if MI principles were referenced or guided design.

^c^Behavioral: observable behavior change (eg, reduced smoking); psychological: internal changes (eg, confidence and readiness); and engagement: user experience, satisfaction, and usability.

^d^NLP: natural language processing.

^e^LLM: large language model.

^f^CEMI: Client Evaluation of Motivational Interviewing.

^g^VR: virtual reality.

^h^ML: machine learning.

## Discussion

### Principal Findings

This scoping review offers a comprehensive synthesis of recent empirical studies evaluating AI-driven systems that simulate MI to promote health behavior change. We identified 15 studies spanning a range of health domains and AI modalities, from simple rule-based chatbots to systems powered by LLMs. While the field remains in its early stages, our review reveals a growing interest in using AI to scale MI-informed interventions and offers insight into feasibility, fidelity, and behavioral impact.

Our findings build on and extend prior reviews of AI in health behavior change interventions; for instance, Kurniawan et al [28] conducted a systematic review examining the use of AI-powered chatbots for chronic disease management and found that those using NLP were generally well accepted by patients, although their clinical effectiveness remains unclear. Similarly, Shingleton and Palfai [29] reviewed digital MI interventions for substance use and concluded that automated MI delivery showed potential but required stronger evidence of behavior change. Pedamallu et al [15] conducted a scoping review focused on chatbots with MI elements, although the authors did not assess fidelity to MI frameworks or focus on AI modality distinctions. Our review focuses specifically on MI-enabled systems and highlights how AI can simulate MI principles, including empathy, evocation, and autonomy support. Compared to broader reviews of conversational agents or digital therapeutics, our synthesis offers a more focused lens on the role of AI in operationalizing MI techniques and advancing behavior change theory in real-world contexts.

One of the most consistent findings across the included studies was the perception of AI systems as nonjudgmental and emotionally neutral. Users in the studies by Brown et al [4], Prochaska et al [7], and Yeo et al [9] explicitly noted feeling more comfortable disclosing stigmatized behaviors such as smoking or alcohol use to a chatbot than to a human provider. Similar perceptions of reduced judgment or increased psychological safety were also reported in the studies by Yoon et al [8], Li et al [11], Shah et al [26], and Park et al [25]. This aligns with MI’s emphasis on reducing resistance and supporting intrinsic motivation through empathetic, nonconfrontational dialogue. Several of the studies (4/15, 27%) reported that participants valued the system’s neutrality, 24/7 availability, and lack of perceived judgment. These are all factors that seemed to foster openness and introspection.

Despite these advantages, limitations in emotional nuance and conversational depth were also frequently noted; for instance, Yoon et al [8] and Yeo et al [9] found that although participants appreciated the usability and structure of AI systems, they often missed the “human touch” and relational complexity typical of face-to-face MI. This trade-off between neutrality and relational depth mirrors findings from other reviews of AI in mental health, where the scalability of digital tools is weighed against limitations in empathetic connection and contextual sensitivity [12,15].

Some of the studies (3/15, 20%) sought to mitigate these limitations by enhancing system interactivity; for example, Kumar et al [5] explored the use of backward-looking reflections or utterances that synthesize earlier user input and guide dialogue based on prior responses. This technique approximates deeper MI engagement by helping users reflect on their own patterns and motivation. Similarly, Steenstra et al [27] used pacing adjustments, or the modulation of response timing and tone, to simulate more natural conversational rhythms. Brown et al [4] used dynamic conversational scaffolding, layering prompts, and affirmations based on prior responses to sustain engagement. These approaches approximate MI’s dialogic rhythm and psychological depth while highlighting areas for technical improvement in LLM-based agents.

MI fidelity, a core component of this review, was formally assessed in 6 (40%) of the 15 studies through methods ranging from expert review to structured rating tools (eg, the MITI and Client Evaluation of Motivational Interviewing scales). Studies such as those by Kumar et al [5] and Herbert et al [6] provided evidence of MI-consistent outputs, while others, such as those by Steenstra et al [27], analyzed conversational transcripts for thematic alignment. However, only a few systems (2/15, 13%) operationalized MI beyond single-turn utterances; for instance, Kumar et al [5] and Herbert et al [6] focused on generating and rating individual MI reflections rather than delivering full-session counseling. This distinction is important: although such systems contribute valuable data on specific MI components, they do not constitute end-to-end MI interventions. These findings reinforce the need to clarify whether systems deliver full MI conversations or produce discrete agent utterances.

Behavioral outcomes were less consistently assessed. Only 3 (20%) of the 15 studies reported statistically significant changes in behavior: Prochaska et al [7] showed reductions in cravings and substance use, Meywirth et al [24] found increased engagement with smoking cessation resources, and He et al [22] reported greater intention to quit among participants exposed to an MI-style chatbot versus a neutral control. Most of the other studies (12/15, 80%) reported only proximal or motivational outcomes such as confidence, engagement, or readiness to change, which, while meaningful, cannot substitute for longer-term behavioral impact. This limitation is echoed in broader AI–behavioral health literature, which often emphasizes feasibility and acceptability over clinical efficacy [12,15].

Several of the studies (5/15, 33%) in this review noted limitations in AI’s emotional nuance and depth of empathy; for example, Yoon et al [8] and Yeo et al [9] described participants’ appreciation for the structure and usability of AI tools but also expressed concerns about the absence of human warmth and relational connection. Li et al [11] similarly acknowledged that users found the digital assistant informative yet somewhat lacking in emotional attunement. Herbert et al [6] and Steenstra et al [27] discussed the challenge of achieving naturalistic dialogue and nuanced responsiveness using current AI models. These findings point to an ongoing difficulty in approximating the relational complexity that characterizes human-delivered MI.

The evidence for the capacity of the MI-based AI systems included in this review to promote behavior change remains limited. While several of the systems (12/15, 80%) demonstrated improvements in motivation, confidence, or readiness to change, only a few of the studies (3/15, 20%), such as those by Prochaska et al [7], Meywirth et al [24], and He et al [22], reported measurable behavioral outcomes. This limitation should not be generalized to all AI behavior change systems. Prior work, including RCTs and systematic reviews focused on digital mental health interventions and chronic disease management [12,30-32], has shown that AI tools can be effective in promoting behavioral change.

Of note, only 3 (20%) of the 15 studies were RCTs [10,22,23]. These RCTs used varied designs, including comparative arms with confrontational counseling or neutral chatbots, to assess the specific value of MI framing. While early results are promising, most of the studies (12/15, 80%) were still in developmental or pilot phases. Given the limited scale and variability in study quality, the findings should be interpreted cautiously.

An additional limitation is the inconsistency in reporting AI system architecture. Several of the studies (5/15, 33%) labeled their tools as chatbots or virtual agents without specifying whether NLP, machine learning, or LLMs were used. This ambiguity hampers replication and prevents nuanced comparisons of system capabilities. Moreover, only a few of the studies (3/15, 20%) addressed safety mechanisms. Furthermore, the use of LLMs for health advice introduces a known risk of generating inaccurate, misleading, or inappropriate content. Even with extensive testing, these models can produce hallucinations or unsafe recommendations, underscoring the importance of transparent safety disclosures and validation protocols in clinical applications [33]. The study by Herbert et al [6] was the only one that explicitly evaluated LLM outputs for both safety and clinical alignment. Future AI-MI applications must incorporate risk mitigation strategies such as disclaimers, real-time oversight, restricted content domains, or integration with human supervision, especially in clinical or behavioral health environments.

In sum, AI-enabled MI systems show strong potential for scaling health behavior interventions, especially in stigmatized or underresourced settings. However, substantial work remains to standardize fidelity assessment, evaluate real-world effectiveness, clarify system design, and balance technical scalability with relational depth.

### Limitations

This review has several limitations. First, although we used a comprehensive search strategy across 5 databases and followed established scoping review methodology, it is possible that relevant studies, particularly those not published in indexed journals or not explicitly labeled as “motivational interviewing,” were missed. Second, we restricted inclusion to English-language studies, which may have excluded relevant research published in other languages. Third, although we included studies that evaluated both full MI interventions and discrete MI components, the heterogeneity in study design, AI modality, and outcome measures limited direct comparison across the included studies. Fourth, several of the included studies (5/15 33%) lacked detailed descriptions of their AI architecture or fidelity assessment protocols, making it difficult to assess methodological rigor. Fifth, despite our efforts to standardize fidelity definitions, interpretations may vary, especially in the absence of validated automated MI coding tools. Finally, most of the studies (12/15, 80%) were early-phase evaluations with small samples, short follow-up periods, and limited generalizability to clinical settings.

### Conclusions

AI-driven systems show growing promise in delivering MI to support health behavior change. Across diverse health contexts and technological platforms, these systems have demonstrated feasibility, user acceptability, and preliminary fidelity to core MI principles such as empathy, collaboration, and evocation. However, only a subset of studies has evaluated behavioral outcomes or used formal fidelity assessments. As interest in AI-enabled health interventions grows, future research should prioritize rigorous evaluation, transparency in AI functionality, and attention to ethical considerations. By combining scalable AI tools with evidence-based behavioral frameworks such as MI, there is significant potential to improve access, engagement, and support for patients navigating complex behavior change.

## Appendix

Multimedia Appendix 1 Search strategy.

Multimedia Appendix 2 Data extraction tool.

Multimedia Appendix 3 Comprehensive overview of studies evaluating AI-based motivational interviewing systems (n = 15). This supplementary table includes detailed characteristics of all included studies, including AI modality and type, motivational interviewing techniques applied, safety considerations, description of study arms, and limitations.

Multimedia Appendix 4 PRISMA-ScR checklist.

## Abbreviations

AI: artificial intelligence
LLM: large language model
MeSH: Medical Subject Headings
MI: motivational interviewing
MITI: Motivational Interviewing Treatment Integrity
NLP: natural language processing
PRISMA-ScR: Preferred Reporting Items for Systematic Reviews and Meta-Analyses extension for Scoping Reviews
RCT: randomized controlled trial
