# Supplementary material for: New Doc on the Block: Scoping Review of AI Systems Delivering Motivational Interviewing for Health Behavior Change
Source: J Med Internet Res. 2025 Sep 16;27:e78417. doi: 10.2196/78417 (PMC12485255; doi:10.2196/78417)
Supplement: Multimedia Appendix 1 [file jmir_v27i1e78417_app1.docx]

**Database: PubMed.gov (Includes MEDLINE)**

(("Artificial Intelligence"[MeSH Terms] OR "Robotics"[MeSH Terms] OR ("adaptive learning"[Title/Abstract] OR "AI"[Title/Abstract] OR "artificial intelligence"[Title/Abstract] OR "app"[Title/Abstract] OR "apps"[Title/Abstract] OR "chatbot*"[Title/Abstract] OR "chatgpt*"[Title/Abstract] OR "chat gpt*"[Title/Abstract] OR "computational intelligence"[Title/Abstract] OR "computer vision system"[Title/Abstract] OR "deep learning"[Title/Abstract] OR "genAI"[Title/Abstract] OR "large language model"[Title/Abstract] OR "machine intelligence"[Title/Abstract] OR "machine learning"[Title/Abstract] OR "robot*"[Title/Abstract] OR "virtual health assistants"[Title/Abstract] OR ("Anthropic"[Title/Abstract] OR "Bard"[Title/Abstract] OR "Bardeen"[Title/Abstract] OR "BERT"[Title/Abstract] OR "Bing chat"[Title/Abstract] OR "BioGPT"[Title/Abstract] OR "BLOOM"[Title/Abstract] OR "chatgpt*"[Title/Abstract] OR "Chat GPT"[Title/Abstract] OR "chatbot*"[Title/Abstract] OR "Chatsonic"[Title/Abstract] OR "Chinchilla"[Title/Abstract] OR "Claude"[Title/Abstract] OR "DALL-E"[Title/Abstract] OR "Ernie"[Title/Abstract] OR "Falcon"[Title/Abstract] OR "Galactica"[Title/Abstract] OR "generative pre trained transformer*"[Title/Abstract] OR "generative pretrained transformer*"[Title/Abstract] OR "GitHub Copilot"[Title/Abstract] OR "GLaM"[Title/Abstract] OR "google* assistant"[Title/Abstract] OR "google* bard"[Title/Abstract] OR "google* gemini"[Title/Abstract] OR "Gopher"[Title/Abstract] OR "GPT-1"[Title/Abstract] OR "GPT-2"[Title/Abstract] OR "gpt 3*"[Title/Abstract] OR "gpt 4*"[Title/Abstract] OR "GPTNeo"[Title/Abstract] OR "gpt j*"[Title/Abstract] OR "IBM Watson"[Title/Abstract] OR "LaMDA"[Title/Abstract] OR "LLaMA"[Title/Abstract] OR "microsoft* bing"[Title/Abstract] OR "microsoft* copilot"[Title/Abstract] OR "Midjourney"[Title/Abstract] OR "Minerva"[Title/Abstract] OR "Nvidia"[Title/Abstract] OR "OpenAI"[Title/Abstract] OR "Open AI"[Title/Abstract] OR "PaLM"[Title/Abstract] OR "PathAI"[Title/Abstract] OR "Perplexity"[Title/Abstract] OR "pre trained transformer*"[Title/Abstract] OR "pretrained transformer*"[Title/Abstract] OR ("apple*"[Title/Abstract] AND "Siri"[Title/Abstract]) OR "Stable Diffusion"[Title/Abstract] OR "StyleGAN"[Title/Abstract] OR "Synthesia"[Title/Abstract] OR "XLNet"[Title/Abstract] OR "100B"[Title/Abstract] OR "YouChat"[Title/Abstract]))) AND ("Motivational interviewing"[MeSH Terms] OR "behavior change counseling"[Title/Abstract] OR "collaborative therapy"[Title/Abstract] OR "motivation enhance*"[Title/Abstract] OR "motivation intervention*"[Title/Abstract] OR "motivation interview*"[Title/Abstract] OR "motivation training"[Title/Abstract] OR "motivational enhance*"[Title/Abstract] OR "motivational intervention*"[Title/Abstract] OR "motivational interview*"[Title/Abstract] OR "motivational training"[Title/Abstract] OR "patient centered counseling"[Title/Abstract])) AND (2018:2025[pdat])

**Database: Embase.com (Elsevier)**

(('Artificial Intelligence'/exp OR 'Artificial Intelligence Chatbot'/exp OR 'Artificial Intelligence Software'/exp OR Robot/exp OR Robotics/exp OR ('adaptive learning':ti,ab OR AI:ti,ab OR 'artificial intelligence':ti,ab OR app:ti,ab OR apps:ti,ab OR chatbot*:ti,ab OR chatgpt*:ti,ab OR 'chat gpt*':ti,ab OR 'computational intelligence':ti,ab OR 'computer vision system':ti,ab OR 'deep learning':ti,ab OR genAI:ti,ab OR 'large language model':ti,ab OR 'machine intelligence':ti,ab OR 'machine learning':ti,ab OR robot*:ti,ab OR 'virtual health assistants':ti,ab OR (Anthropic:ti,ab OR Bard:ti,ab OR Bardeen:ti,ab OR BERT:ti,ab OR 'Bing chat':ti,ab OR BioGPT:ti,ab OR BLOOM:ti,ab OR chatgpt*:ti,ab OR 'Chat GPT':ti,ab OR chatbot*:ti,ab OR Chatsonic:ti,ab OR Chinchilla:ti,ab OR Claude:ti,ab OR DALL-E:ti,ab OR Ernie:ti,ab OR Falcon:ti,ab OR Galactica:ti,ab OR 'generative pre trained transformer*':ti,ab OR 'generative pretrained transformer*':ti,ab OR 'GitHub Copilot':ti,ab OR GLaM:ti,ab OR 'google* assistant':ti,ab OR 'google* bard':ti,ab OR 'google* gemini':ti,ab OR Gopher:ti,ab OR GPT-1:ti,ab OR GPT-2:ti,ab OR 'gpt 3*':ti,ab OR 'gpt 4*':ti,ab OR GPTNeo:ti,ab OR 'gpt j*':ti,ab OR 'IBM Watson':ti,ab OR LaMDA:ti,ab OR LLaMA:ti,ab OR 'microsoft* bing':ti,ab OR 'microsoft* copilot':ti,ab OR Midjourney:ti,ab OR Minerva:ti,ab OR Nvidia:ti,ab OR OpenAI:ti,ab OR 'Open AI':ti,ab OR PaLM:ti,ab OR PathAI:ti,ab OR Perplexity:ti,ab OR 'pre trained transformer*':ti,ab OR 'pretrained transformer*':ti,ab OR (apple*:ti,ab AND Siri:ti,ab) OR 'Stable Diffusion':ti,ab OR StyleGAN:ti,ab OR Synthesia:ti,ab OR XLNet:ti,ab OR 100B:ti,ab OR YouChat:ti,ab))) AND ('Motivational interviewing'/exp OR 'behavior change counseling':ti,ab OR 'collaborative therapy':ti,ab OR 'motivation enhance*':ti,ab OR 'motivation intervention*':ti,ab OR 'motivation interview*':ti,ab OR 'motivation training':ti,ab OR 'motivational enhance*':ti,ab OR 'motivational intervention*':ti,ab OR 'motivational interview*':ti,ab OR 'motivational training':ti,ab OR 'patient centered counseling':ti,ab)) AND (2018:py OR 2019:py OR 2020:py OR 2021:py OR 2022:py OR 2023:py OR 2024:py OR 2025:py)

**Database: Cochrane Library**

([mh "Artificial Intelligence"] OR [mh Robotics] OR "adaptive learning":ti,ab OR AI:ti,ab OR "artificial intelligence":ti,ab OR app:ti,ab OR apps:ti,ab OR chatbot*:ti,ab OR chatgpt*:ti,ab OR ("chat" NEXT gpt*):ti,ab OR "computational intelligence":ti,ab OR "computer vision system":ti,ab OR "deep learning":ti,ab OR genAI:ti,ab OR "large language model":ti,ab OR "machine intelligence":ti,ab OR "machine learning":ti,ab OR robot*:ti,ab OR "virtual health assistants":ti,ab OR Anthropic:ti,ab OR Bard:ti,ab OR Bardeen:ti,ab OR BERT:ti,ab OR "Bing chat":ti,ab OR BioGPT:ti,ab OR BLOOM:ti,ab OR chatgpt*:ti,ab OR "Chat GPT":ti,ab OR chatbot*:ti,ab OR Chatsonic:ti,ab OR Chinchilla:ti,ab OR Claude:ti,ab OR DALL-E:ti,ab OR Ernie:ti,ab OR Falcon:ti,ab OR Galactica:ti,ab OR ("generative pre trained" NEXT transformer*):ti,ab OR ("generative pretrained" NEXT transformer*):ti,ab OR "GitHub Copilot":ti,ab OR GLaM:ti,ab OR (google* NEXT "assistant"):ti,ab OR (google* NEXT "bard"):ti,ab OR (google* NEXT "gemini"):ti,ab OR Gopher:ti,ab OR GPT-1:ti,ab OR GPT-2:ti,ab OR ("gpt" NEXT 3*):ti,ab OR ("gpt" NEXT 4*):ti,ab OR GPTNeo:ti,ab OR ("gpt" NEXT j*):ti,ab OR "IBM Watson":ti,ab OR LaMDA:ti,ab OR LLaMA:ti,ab OR (microsoft* NEXT "bing"):ti,ab OR (microsoft* NEXT "copilot"):ti,ab OR Midjourney:ti,ab OR Minerva:ti,ab OR Nvidia:ti,ab OR OpenAI:ti,ab OR "Open AI":ti,ab OR PaLM:ti,ab OR PathAI:ti,ab OR Perplexity:ti,ab OR ("pre trained" NEXT transformer*):ti,ab OR ("pretrained" NEXT transformer*):ti,ab OR (apple*:ti,ab AND Siri:ti,ab) OR "Stable Diffusion":ti,ab OR StyleGAN:ti,ab OR Synthesia:ti,ab OR XLNet:ti,ab OR 100B:ti,ab OR YouChat:ti,ab) AND ([mh "Motivational interviewing"] OR "behavior change counseling":ti,ab OR "collaborative therapy":ti,ab OR ("motivation" NEXT enhance*):ti,ab OR ("motivation" NEXT intervention*):ti,ab OR ("motivation" NEXT interview*):ti,ab OR "motivation training":ti,ab OR ("motivational" NEXT enhance*):ti,ab OR ("motivational" NEXT intervention*):ti,ab OR ("motivational" NEXT interview*):ti,ab OR "motivational training":ti,ab OR "patient centered counseling":ti,ab)

**Database: Web of Science**

(((TI="adaptive learning" OR AB="adaptive learning") OR (TI=AI OR AB=AI) OR (TI="artificial intelligence" OR AB="artificial intelligence") OR (TI=app OR AB=app) OR (TI=apps OR AB=apps) OR (TI=chatbot* OR AB=chatbot*) OR (TI=chatgpt* OR AB=chatgpt*) OR (TI="chat gpt*" OR AB="chat gpt*") OR (TI="computational intelligence" OR AB="computational intelligence") OR (TI="computer vision system" OR AB="computer vision system") OR (TI="deep learning" OR AB="deep learning") OR (TI=genAI OR AB=genAI) OR (TI="large language model" OR AB="large language model") OR (TI="machine intelligence" OR AB="machine intelligence") OR (TI="machine learning" OR AB="machine learning") OR (TI=robot* OR AB=robot*) OR (TI="virtual health assistants" OR AB="virtual health assistants") OR (TI=Anthropic OR AB=Anthropic) OR (TI=Bard OR AB=Bard) OR (TI=Bardeen OR AB=Bardeen) OR (TI=BERT OR AB=BERT) OR (TI="Bing chat" OR AB="Bing chat") OR (TI=BioGPT OR AB=BioGPT) OR (TI=BLOOM OR AB=BLOOM) OR (TI=chatgpt* OR AB=chatgpt*) OR (TI="Chat GPT" OR AB="Chat GPT") OR (TI=chatbot* OR AB=chatbot*) OR (TI=Chatsonic OR AB=Chatsonic) OR (TI=Chinchilla OR AB=Chinchilla) OR (TI=Claude OR AB=Claude) OR (TI=DALL-E OR AB=DALL-E) OR (TI=Ernie OR AB=Ernie) OR (TI=Falcon OR AB=Falcon) OR (TI=Galactica OR AB=Galactica) OR (TI="generative pre trained transformer*" OR AB="generative pre trained transformer*") OR (TI="generative pretrained transformer*" OR AB="generative pretrained transformer*") OR (TI="GitHub Copilot" OR AB="GitHub Copilot") OR (TI=GLaM OR AB=GLaM) OR (TI="google* assistant" OR AB="google* assistant") OR (TI="google* bard" OR AB="google* bard") OR (TI="google* gemini" OR AB="google* gemini") OR (TI=Gopher OR AB=Gopher) OR (TI=GPT-1 OR AB=GPT-1) OR (TI=GPT-2 OR AB=GPT-2) OR (TI="gpt 3*" OR AB="gpt 3*") OR (TI="gpt 4*" OR AB="gpt 4*") OR (TI=GPTNeo OR AB=GPTNeo) OR (TI="gpt j*" OR AB="gpt j*") OR (TI="IBM Watson" OR AB="IBM Watson") OR (TI=LaMDA OR AB=LaMDA) OR (TI=LLaMA OR AB=LLaMA) OR (TI="microsoft* bing" OR AB="microsoft* bing") OR (TI="microsoft* copilot" OR AB="microsoft* copilot") OR (TI=Midjourney OR AB=Midjourney) OR (TI=Minerva OR AB=Minerva) OR (TI=Nvidia OR AB=Nvidia) OR (TI=OpenAI OR AB=OpenAI) OR (TI="Open AI" OR AB="Open AI") OR (TI=PaLM OR AB=PaLM) OR (TI=PathAI OR AB=PathAI) OR (TI=Perplexity OR AB=Perplexity) OR (TI="pre trained transformer*" OR AB="pre trained transformer*") OR (TI="pretrained transformer*" OR AB="pretrained transformer*") OR ((TI=apple* OR AB=apple*) AND (TI=Siri OR AB=Siri)) OR (TI="Stable Diffusion" OR AB="Stable Diffusion") OR (TI=StyleGAN OR AB=StyleGAN) OR (TI=Synthesia OR AB=Synthesia) OR (TI=XLNet OR AB=XLNet) OR (TI=100B OR AB=100B) OR (TI=YouChat OR AB=YouChat)) AND ((TI="behavior change counseling" OR AB="behavior change counseling") OR (TI="collaborative therapy" OR AB="collaborative therapy") OR (TI="motivation enhance*" OR AB="motivation enhance*") OR (TI="motivation intervention*" OR AB="motivation intervention*") OR (TI="motivation interview*" OR AB="motivation interview*") OR (TI="motivation training" OR AB="motivation training") OR (TI="motivational enhance*" OR AB="motivational enhance*") OR (TI="motivational intervention*" OR AB="motivational intervention*") OR (TI="motivational interview*" OR AB="motivational interview*") OR (TI="motivational training" OR AB="motivational training") OR (TI="patient centered counseling" OR AB="patient centered counseling")) AND ((TI="behavior change counseling" OR AB="behavior change counseling") OR (TI="collaborative therapy" OR AB="collaborative therapy") OR (TI="motivation enhance*" OR AB="motivation enhance*") OR (TI="motivation intervention*" OR AB="motivation intervention*") OR (TI="motivation interview*" OR AB="motivation interview*") OR (TI="motivation training" OR AB="motivation training") OR (TI="motivational enhance*" OR AB="motivational enhance*") OR (TI="motivational intervention*" OR AB="motivational intervention*") OR (TI="motivational interview*" OR AB="motivational interview*") OR (TI="motivational training" OR AB="motivational training") OR (TI="patient centered counseling" OR AB="patient centered counseling"))))

**Database: Scopus**

( ( INDEXTERMS ( "Artificial Intelligence" ) OR INDEXTERMS ( robotics ) OR ( TITLE-ABS ( "adaptive learning" ) OR TITLE-ABS ( ai ) OR TITLE-ABS ( "artificial intelligence" ) OR TITLE-ABS ( app ) OR TITLE-ABS ( apps ) OR TITLE-ABS ( chatbot* ) OR TITLE-ABS ( chatgpt* ) OR TITLE-ABS ( "chat gpt*" ) OR TITLE-ABS ( "computational intelligence" ) OR TITLE-ABS ( "computer vision system" ) OR TITLE-ABS ( "deep learning" ) OR TITLE-ABS ( genai ) OR TITLE-ABS ( "large language model" ) OR TITLE-ABS ( "machine intelligence" ) OR TITLE-ABS ( "machine learning" ) OR TITLE-ABS ( robot* ) OR TITLE-ABS ( "virtual health assistants" ) OR ( TITLE-ABS ( anthropic ) OR TITLE-ABS ( bard ) OR TITLE-ABS ( bardeen ) OR TITLE-ABS ( bert ) OR TITLE-ABS ( "Bing chat" ) OR TITLE-ABS ( biogpt ) OR TITLE-ABS ( bloom ) OR TITLE-ABS ( chatgpt* ) OR TITLE-ABS ( "Chat GPT" ) OR TITLE-ABS ( chatbot* ) OR TITLE-ABS ( chatsonic ) OR TITLE-ABS ( chinchilla ) OR TITLE-ABS ( claude ) OR TITLE-ABS ( dall-e ) OR TITLE-ABS ( ernie ) OR TITLE-ABS ( falcon ) OR TITLE-ABS ( galactica ) OR TITLE-ABS ( "generative pre trained transformer*" ) OR TITLE-ABS ( "generative pretrained transformer*" ) OR TITLE-ABS ( "GitHub Copilot" ) OR TITLE-ABS ( glam ) OR TITLE-ABS ( "google* assistant" ) OR TITLE-ABS ( "google* bard" ) OR TITLE-ABS ( "google* gemini" ) OR TITLE-ABS ( gopher ) OR TITLE-ABS ( gpt-1 ) OR TITLE-ABS ( gpt-2 ) OR TITLE-ABS ( "gpt 3*" ) OR TITLE-ABS ( "gpt 4*" ) OR TITLE-ABS ( gptneo ) OR TITLE-ABS ( "gpt j*" ) OR TITLE-ABS ( "IBM Watson" ) OR TITLE-ABS ( lamda ) OR TITLE-ABS ( llama ) OR TITLE-ABS ( "microsoft* bing" ) OR TITLE-ABS ( "microsoft* copilot" ) OR TITLE-ABS ( midjourney ) OR TITLE-ABS ( minerva ) OR TITLE-ABS ( nvidia ) OR TITLE-ABS ( openai ) OR TITLE-ABS ( "Open AI" ) OR TITLE-ABS ( palm ) OR TITLE-ABS ( pathai ) OR TITLE-ABS ( perplexity ) OR TITLE-ABS ( "pre trained transformer*" ) OR TITLE-ABS ( "pretrained transformer*" ) OR ( TITLE-ABS ( apple* ) AND TITLE-ABS ( siri ) ) OR TITLE-ABS ( "Stable Diffusion" ) OR TITLE-ABS ( stylegan ) OR TITLE-ABS ( synthesia ) OR TITLE-ABS ( xlnet ) OR TITLE-ABS ( 100b ) OR TITLE-ABS ( youchat ) ) ) ) AND ( INDEXTERMS ( "Motivational interviewing" ) OR TITLE-ABS ( "behavior change counseling" ) OR TITLE-ABS ( "collaborative therapy" ) OR TITLE-ABS ( "motivation enhance*" ) OR TITLE-ABS ( "motivation intervention*" ) OR TITLE-ABS ( "motivation interview*" ) OR TITLE-ABS ( "motivation training" ) OR TITLE-ABS ( "motivational enhance*" ) OR TITLE-ABS ( "motivational intervention*" ) OR TITLE-ABS ( "motivational interview*" ) OR TITLE-ABS ( "motivational training" ) OR TITLE-ABS ( "patient centered counseling" ) ) ) AND PUBYEAR > 2017 AND PUBYEAR < 2026

**Date Limiter: 2018 - 2025**

**Note:** Artificial Intelligence search was partially adapted from University of Alberta Filters to Retrieve Studies Related to Generative AI at <https://docs.google.com/document/d/1fPiY7GmI_Z4xZQTqZ58DySUTJousLlZr1GTCP-kDOLA/edit?tab=t.0>
