# Supplementary material for: New Doc on the Block: Scoping Review of AI Systems Delivering Motivational Interviewing for Health Behavior Change
Source: J Med Internet Res. 2025 Sep 16;27:e78417. doi: 10.2196/78417 (PMC12485255; doi:10.2196/78417)
Supplement: Multimedia Appendix 2 [file jmir_v27i1e78417_app2.docx]

**Appendix II: Data Extraction Instrument**

**General information**

- Study ID
- Study Title
- Authors
- Year of Publication
- Country
- Notable contextual information (e.g., funding, conflicts of interest)

**Study Characteristics**

- Study Aim / Research Objective
- Study design:
- Randomized controlled trial
- Feasibility/Usability Study
- Exploratory/Descriptive
- Cohort study/Observational
- Qualitative study
- Other

**Intervention Details**

- AI Modality (e.g., chatbot, mobile app, VR agent)
- AI Type (e.g., rule-based, LLM-based, hybrid)
- MI Techniques Implemented (check all that apply):

| **MI Technique** | **Description** |
| --- | --- |
| Open-ended questions | Questions that invite elaboration and avoid yes/no answers |
| Affirmations | Statements recognizing patient strengths or efforts |
| Reflections | Rephrasing or summarizing patient statements to convey understanding |
| Ruler Talk | Asking patients to rate readiness/confidence/importance on a numeric scale |
| Change Talk Elicitation | Prompting statements that favor behavior change |

**MI Fidelity Assessment**

- Was MI fidelity assessed?
  - Yes / No
- If yes, how was fidelity assessed? (check all that apply):
- Expert reviewer rating
- Structured coding framework (e.g., MITI, MISC)
- Automated NLP evaluation
- Self-reported by participants
- Other (specify): _________
- What MI components were assessed for fidelity?
- Empathy
- Reflective listening
- Open-ended questions
- Change talk elicitation
- Collaboration / Autonomy support
- Other: __________
- Summary of MI fidelity findings (if applicable):

**Study Comparison Variables**

- Independent Variable(s) / Intervention Features Tested
- Was there a Comparison Group?
  - Yes / No
  - If yes, describe (e.g., human-delivered MI, non-AI chatbot)
- Dependent Variable(s) / Outcome(s) Measured
- Outcome Domain (select one):
  - Behavioral
  - Psychological / Cognitive
  - Engagement / Usability
  - Clinical
  - Not Specified

**Participants**

- Population description (e.g., general adults, specific condition, underserved group)
- Targeted Health Behavior (check all that apply):
- Substance use cessation
- Exercise
- Nutrition
- Vaccine uptake
- Chronic disease management
- Other
- Total number of participants:

**Findings and Interpretation**

- Key Findings:
- Was the AI-delivered MI perceived as effective by participants?
   Yes  No  Not Reported
- Were comparisons made to human MI or non-AI versions?
   Yes  No  Not Applicable
  If yes, describe:
- How did AI-MI compare in terms of fidelity, engagement, or outcomes?
- **Addressed Safety?** Reflect whether the study explicitly addressed safety concerns such as the use of expert validation, acknowledgment of risks with LLMs, or the absence of safety disclosures.

**Limitations and Conclusions**

- Author-Reported Limitations:
- Reviewer-Identified Limitations:
- Summary of Authors’ Conclusions:

**Supplementary Glossary**

| **Term** | **Definition** |
| --- | --- |
| Dynamic conversational scaffolding | A conversational design strategy where AI builds contextually on prior responses to personalize support and maintain dialogue flow. |
| Backward-looking reflections | Statements that summarize or rephrase earlier user input to convey empathy and reinforce understanding. |
| Pacing adjustments | Modulating the speed, timing, or complexity of dialogue to match user engagement and cognitive load. |
